# Building Mutually Beneficial Collaborations Between Digital Navigators, Mental Health Professionals, and Clients: Naturalistic Observational Case Study

**DOI:** 10.2196/58068

**Published:** 2024-11-06

**Authors:** Carla Gorban, Sarah McKenna, Min K Chong, William Capon, Robert Battisti, Alison Crowley, Bradley Whitwell, Antonia Ottavio, Elizabeth M Scott, Ian B Hickie, Frank Iorfino

**Affiliations:** 1 Brain and Mind Centre, The University of Sydney, 94 Mallett Street, Sydney, 2050, Australia, 61 (02) 9351 0774; 2 Mind Plasticity, Sydney, Australia; 3 Headspace Bondi Junction, Sydney, Australia

**Keywords:** digital navigator, digital coach, clinical technology specialist, mental health services, shared decision-making, lived experience, implementation, poor engagement, decision-making, mental health, digital mental health, digital mental health technology

## Abstract

Despite the efficacy of digital mental health technologies (DMHTs) in clinical trials, low uptake and poor engagement are common in real-world settings. Accordingly, digital technology experts or “digital navigators” are increasingly being used to enhance engagement and shared decision-making between health professionals and clients. However, this area is relatively underexplored and there is a lack of data from naturalistic settings. In this paper, we report observational findings from the implementation of a digital navigator in a multidisciplinary mental health clinic in Sydney, Australia. The digital navigator supported clients and health professionals to use a measurement-based DMHT (the Innowell platform) for improved multidimensional outcome assessment and to guide personalized decision-making. Observational data are reported from implementation logs, platform usage statistics, and response rates to digital navigator emails and phone calls. Ultimately, support from the digital navigator led to improved data collection and clearer communications about goals for using the DMHT to track client outcomes; however, this required strong partnerships between health professionals, the digital navigator, and clients. The digital navigator helped to facilitate the integration of DMHT into care, rather than providing a stand-alone service. Thus, collaborations between health professionals and digital navigators are mutually beneficial and empower clients to be more engaged in their own care.

## Introduction

Although the demand for care has been rising, mental health care systems are falling short, with most services reporting high wait times for care, high dropout rates, and difficulties providing access to specialized care [1,2]. Digital mental health technologies (DMHTs) have the potential to facilitate highly personalized care through multidimensional assessment and more efficient care coordination [3-5]. However, implementation studies have consistently reported low uptake in real-world settings [6-8]. The barriers appear to be multifaceted, as they can be linked to individual attitudes or beliefs toward the technology, the nature of clinical practice, as well as technology-driven or organizational barriers [9-11]. Innovative solutions are needed to ensure that DMHTs can be successfully deployed into real-world settings and improve equitable access to effective mental health treatments.

Given that the barriers are complex and multifaceted, integrating DMHTs in real-world settings requires broad transformations to occur within mental health services. This calls for new supports to be integrated in clinics that can provide better training and assistance to both health professionals and clients. One solution has been the use of health technology experts in real-world services. These experts have been given various titles including “digital coaches,” “clinical technology specialists,” and “digital navigators”—for simplicity, this paper will refer to them as digital navigators [12-14]. Digital navigators can provide unique value to clinical teams by doing the following: (1) evaluating apps so health professionals have confidence recommending useful apps to clients; (2) offering nonclinical technical support, such as troubleshooting between sessions to improve ease of use; and (3) helping to interpret app data before visits and highlighting salient data features to improve the usefulness and clinical value of the technology to health professionals [12]. Digital navigators can expand on more traditional positions, such as community health workers or social workers, to include assisting with the use of new clinical technologies and how to utilize them in care. Moreover, digital navigators can build rapport with clients around the use of DMHTs, which is key to improving engagement and trust in any clinical intervention [12]. Overall, this ensures that health professionals and clients are actively supported to make the best use of digital technologies in health care.

Despite the promise of this approach, the implementation of digital navigators in previous research has been mixed, and there is still limited information about protocols or training [15,16]. Previous work has utilized both clinical and nonclinical staff, used scheduled and on-demand support, and trialed both face-to-face and remote interactions, with similar results [15,16]. A particularly important question to address is whether these roles should be filled by existing members of the care team, or whether a “social coaching model” (to use a term coined by Meyer, Wisniewski, and Torous [15]), which uses peer workers to encourage client engagement and provide support, is more valuable. A review of 26 DMHT trials found only 3 studies that had used a peer worker rather than a health professional, suggesting that the potential benefits of this approach are underexplored [14].

Additionally, the focus of digital navigation in existing literature is most often to support the use of self-guided online therapy modules. Another potential role of digital navigators is to support the integration of digital technologies into traditional therapies provided by health professionals. For example, measurement-based DMHTs aim to improve the identification and tracking of mental disorders over the course of care, ensuring clients receive more appropriate treatments earlier in their illness trajectory [17-19]. These platforms require health professionals to adopt new practices and processes, which often involves proactively responding to new information provided by DMHTs. Past work has consistently found that health professionals need proper training around how to use and interact with DMHTs during sessions for them to be implemented [14]. Accordingly, further work is needed to understand how digital navigators can be integrated in health services and used to enhance existing care options, such as improved outcome monitoring.

This paper presents observational evidence from the real-world implementation of a digital navigator in a multidisciplinary clinic in Sydney, Australia. This role was implemented to support the use of a measurement-based DMHT (the Innowell platform) as part of the EMPOWERED (Educate, Measurement-based, Personalised, Openness, Work collaboratively, Engage, Recovery, Enhanced Digitally) trial, a randomized controlled trial [20]. Specifically, we will focus on the period leading up to the trial in which this role was implemented and tested to ensure feasibility and viability. Although the digital navigator role has been described in our published protocol [20], we aim to provide a more detailed overview of our real-world experiences integrating this role into existing clinical practice to identify valuable, generalizable, and practical insights that could inform future efforts to utilize this role in health services.

## Setting and Background

**Figure 1.**
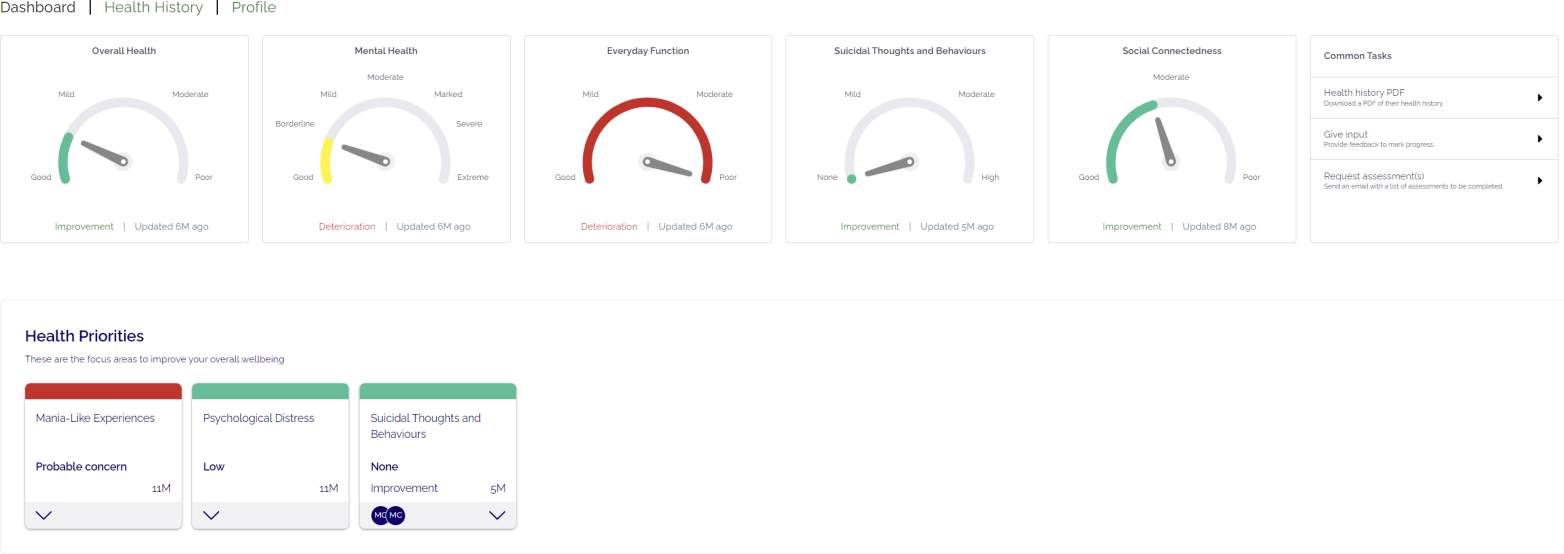
Clinician view of client’s dashboard in Innowell.

**Figure 2.**
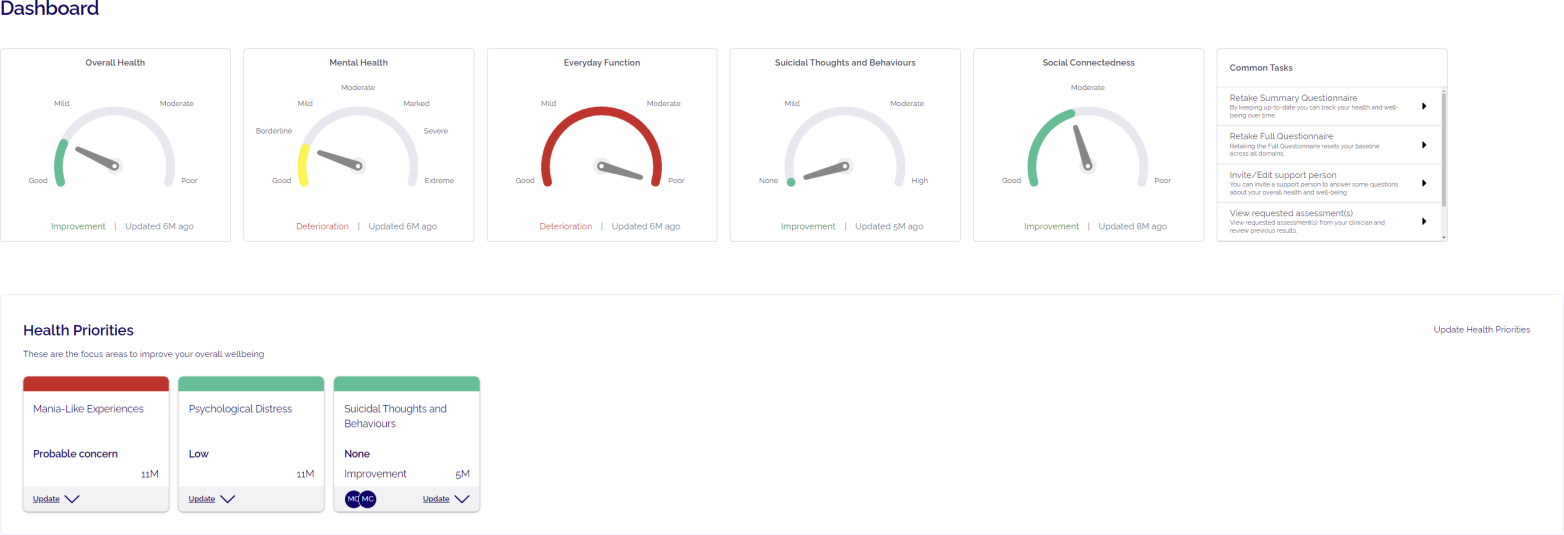
Client view of their own dashboard in Innowell.

**Figure 3.**
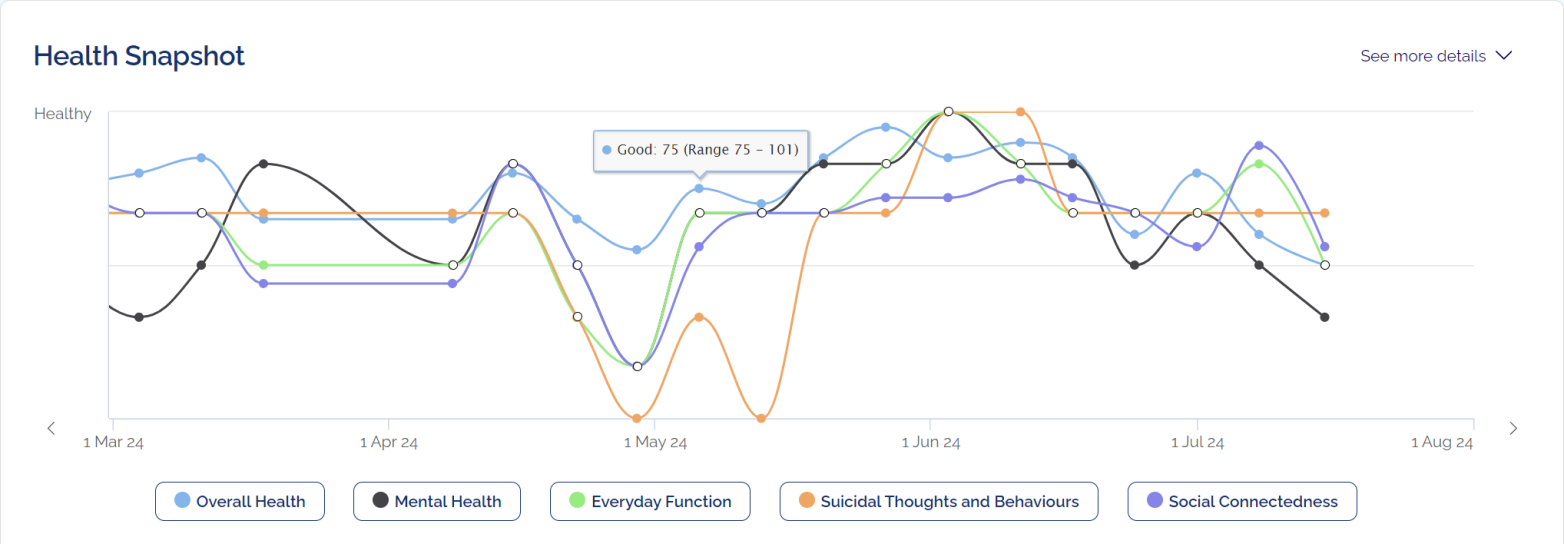
The longitudinal graph of the dashboard available to both clinician and client in Innowell.

The digital navigator was introduced to Mind Plasticity (a private multidisciplinary mental health clinic in Sydney, Australia) in September 2022 to improve uptake of the Innowell platform ahead of the EMPOWERED clinical trial. Mind Plasticity is comprised of 2 clinics servicing approximately 2600 clients, and offers a range of services including psychiatry, psychology, occupational therapy, and education support, as well as a mental health nurse to provide medication management. Innowell was already being used at the service prior to the digital navigator starting. The purpose of Innowell is to facilitate routine outcome monitoring by allowing clients to complete online assessments across a range of domains, including mental and physical health, functioning, substance and alcohol use, and suicidality or self-harm, to track progress in care and guide personalized decision-making (Figures 1-3) [4,19,21]. Previous work has shown that the implementation of DMHTs (such as Innowell) in real-world settings is often limited by an unwillingness to adapt clinical practice to integrate DMHTs [22-24]. As such, there was a need to improve the integration of Innowell within the service ahead of the trial launch. Prior to the trial starting, the digital navigator (author CG) was brought in to help the service establish the system processes and best practices around using Innowell. The goal of this “pretrial” phase was to increase uptake of the platform with service stakeholders (clients and health professionals), assist with the onboarding process to the platform, enhance client engagement with Innowell, and improve overall service integration. The digital navigator continued to play a key role in supporting use of the platform during the trial as discussed in the published protocol [20].

Author CG was recruited to fill the role of digital navigator in the clinical trial, due to both her lived and professional expertise. Our team sought a person with lived experience of mental ill-health to fill the role of digital navigator so that this role would be more accessible and relatable to clients. Additionally, peer workers or those with lived experience are well placed in the role of digital navigators as their lived experience provides a unique perspective to walk side-by-side with a young person throughout their care-journey. CG also had professional experience from a previous implementation, which informed the current approach. This process has been outlined in detail in previous publications [4,23].

## Observational Data Collection

Observational logs were used by the digital navigator to capture qualitative data about the role, including interactions with health professionals and clients (Table 1). These logs were based on feedback from face-to-face interactions, phone calls, and emails. The digital navigator also tracked client engagement with the Innowell platform and frequency of responses to calls and emails to gain further information about engagement.

**Table 1.** Observational findings from digital navigator logs on the workability and usefulness of our processes.

	What did not work?	What did?
First contact with digital navigator	Health professionals did not have time between appointments to introduce clients to the digital navigator in person, partly because they often worked off-site. Many clients preferred telehealth sessions and did not attend the clinic in person.	Health professionals provided a list of clients for the digital navigator to follow up with, who had already agreed to be contacted. One health professional made time after each session to introduce the client to the digital navigator and discuss goals for using the platform together.
Onboarding clients to the platform	All clients were reinvited to the platform to improve service uptake, and the digital navigator sent a 2-week reminder. Health professionals believed it was the digital navigator’s role to introduce clients to the platform.	The digital navigator sent follow-up reminders 2 days before an appointment. Health professionals introduced the platform during the session and asked the digital navigator to provide follow-up support.
Improving ongoing use of the platform	Clients did not generally seek ad hoc support between appointments from the digital navigator through face-to-face meetings. Health professionals believed that clients would let them know if they wanted to discuss data from the platform in their care. Digital navigator encouraged clients to use platform and to take initiative in asking the health professional to discuss data from the platform.	Clients preferred to schedule communication at a time that worked for them and to choose the method (ie, emails, phone calls, Zoom, or face to face) and frequency of this communication. Health professionals had frequent communication with the young person and digital navigator to discuss what outcomes were being tracked. Digital navigator showed the young person the functionalities of the platform and helped them identify features that were valuable to them to assist them to get the most use out of the platform.

Principles of thematic analysis were used to identify common problems and solutions associated with the digital navigator role and its value in health services [25]. Preliminary analysis was conducted by the primary coder (CG), who read through the observational logs and identified common themes across the case studies. These themes were discussed with 2 secondary coders, a clinical academic researcher (colead author SM) and a senior academic researcher (author FI). CG then reviewed the case studies and identified those that reflected the main themes and that illustrated the variability in how the digital navigator role can be experienced. The case studies were then discussed with the secondary coders and the final cases and themes were agreed upon.

Principles of constructionist theory grounded our analysis, which argues that all knowledge is constructed through the experiences and subjectivities that researchers bring to the data. CG has lived experience and is a digital navigator with expertise regarding contextual factors, and SM is a clinical psychologist and is experienced in cognitive behavioral therapies that emphasize interrelationships between attitudes, experiences, and behaviors. FI is experienced in implementation of digital technologies and has knowledge of common systemic and individual-level factors that influence uptake of new technologies. These preexisting subjectivities shaped our interpretations of the data.

## Description of Digital Navigator Role

**Figure 4.**
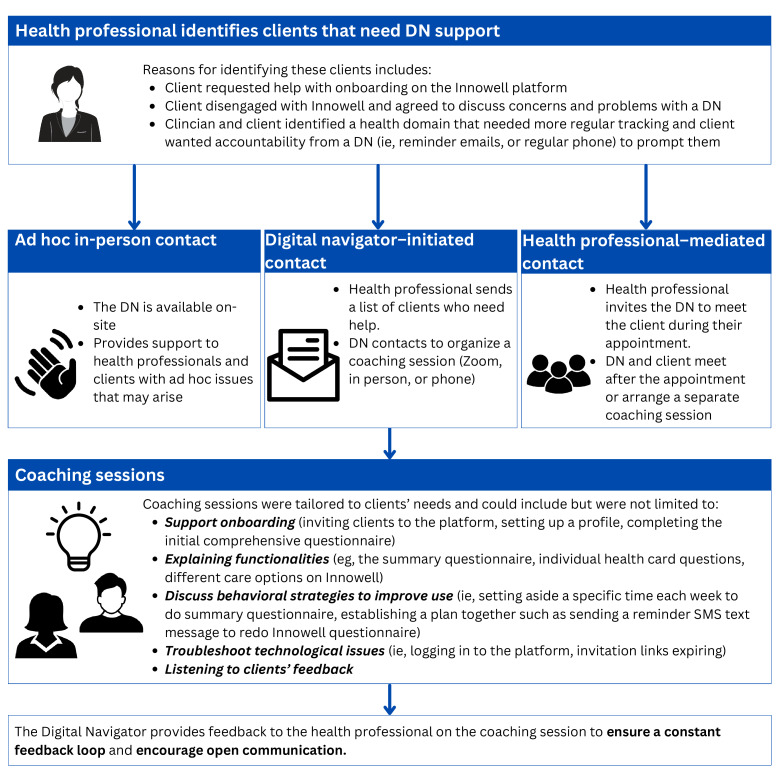
Overview of referral process and purpose of digital navigator sessions. DN: digital navigator.

Figure 4 is a detailed description of the referral process and purpose of coaching sessions. A referral-style process was used, where a health professional identified a client who would benefit from the digital navigator’s support. The digital navigator then arranged unstructured “digital navigator sessions” that were tailored to the needs of the individual client. In total, there were 25 digital navigator sessions over Zoom or in-person at the clinics. The average length of time was 30 minutes per session and the digital navigator saw a range of clients, including those with attention-deficit/hyperactivity disorder and autism spectrum disorder; mood disorders such as bipolar disorder and depression; and substance misuse. The digital navigator provided feedback on the session to the health professional to ensure a constant feedback loop and encourage open communication.

Initial meetings would be used to establish rapport with the client and introduce the Innowell platform and its functions, and provide training and guidance on how to use the platform regularly (ie, completing the summary questionnaire every 1‐2 weeks, before appointments with a health professional). During this visit, the digital navigator and the client would discuss the benefits of personalized and measurement-based care, and how Innowell can be used to support this care approach. A personalized plan would then be devised to support ongoing engagement with Innowell and its use in care. During this meeting, clients were asked how they would like to be contacted, how frequently, and for what purpose. For example, some clients preferred receiving reminder SMS text messages a couple of days before their appointment at the service, whereas other clients preferred email reminders at a set time (eg, fortnightly, on Mondays). Ongoing communication was mostly flexible and tailored to the needs of the individual. Figure 4 describes several activities that took place during these sessions.

## Findings From Observational Data

Table 1 provides a summary of the aspects of the digital navigator role that were and were not successful for increasing engagement with the Innowell platform. We have summarized our experiences in more detail below.

### Types of Engagement

The value of the digital navigator was not necessarily observed for increasing overall uptake of the platform but rather for improving the perceived usefulness of Innowell. Over a 2-month test period (February-April 2023), the digital navigator sent 235 emails to 204 individual clients of 4 senior health professionals, including 2 psychiatrists and 2 clinical psychologists, as part of a large-scale engagement activity. This involved a reminder to complete the onboarding process and initial Innowell questionnaire prior to their appointments at the service. However, only 18% (37/204) completed the questionnaire from this prompt by the digital navigator.

Notably, the nature and frequency of interactions between the digital navigator and clinicians seemed to influence clients’ engagement with the Innowell platform and the type of contact with the digital navigator was important. Some health professionals invited the digital navigator to sessions and facilitated an introduction, whereas other clients were only contacted by email. One health professional, Clinician A, who had engaged the digital navigator as part of their care team, had a lower rate of expired links than the other clinicians, who did not have frequent contact with the digital navigator. Expired links occur when a client has not engaged with the onboarding email within 7 days. This clinician had just 5% of clients with expired links, whereas the average for other clinicians was 35%‐43%. Thus, the clients who were most likely to fill out questionnaires on the platform after contact from the digital navigator were those who had health professionals that strongly promoted using the DMHT and engaging with the digital navigator.

This is further demonstrated by case studies 1 and 2. Case study 1 (Textbox 1) describes how a client who had never before used the Innowell platform in their care had a positive experience meeting with the digital navigator and appeared to be engaged for several weeks; however, they eventually stopped using the platform. By contrast, case study 2 (Textbox 2) describes how the digital navigator supported the client to communicate their concerns to their health professional and ensured that the DMHT was used more frequently in care according to the client’s wishes. In this way, digital navigators can empower clients to make shared decisions about the use of the DMHT in their care and even strengthen therapeutic relationships.

In other words, implementing digital navigators in services is not enough to improve uptake. The frequency with which clients use DMHT is most likely to depend on health professionals’ attitudes and behaviors. Instead, the digital navigator can remove some of the burdens of integrating new technology in care and help both clients and health professionals to understand how the technology can be integrated effectively.

Textbox 1. **Case study 1: The digital navigator may not improve long-term engagement when working in isolation.**

**Client details**

The client and their psychologist had discussed using Innowell to help monitor mood and their symptoms and the client needed support with the onboarding process to the platform and how to use the technology. The client had never used Innowell before.

**Initial session with the digital navigator**

The digital navigator met with the client for roughly 1 hour via Zoom. The digital navigator assisted the client with the onboarding process (accessing secure invite links; creating and saving profiles; bookmarking the login page to their web browser, etc). The client asked questions such as how to use Innowell in sessions with their health professional and how frequently they should answer the summary questionnaire.

The digital navigator explained the purpose of the platform—how their psychologist can review their Innowell dashboard prior to appointments, making sessions more tailored to the client’s unique changes and experiences based on the client’s data inputs in the platform. The client and clinician can then review Innowell data together during appointments by having the client’s Innowell dashboard open on the computer screen in the consult room. The digital navigator explained that answering the summary questionnaire weekly to fortnightly could be valuable as it coincided with the client’s regular appointments.

At the end of the coaching session, the client said they felt confident using the technology and requested the digital navigator send text message reminders weekly to complete the summary questionnaire before attending their appointment with the psychologist. The client requested this method of contact as it would be like having “an accountability buddy,” and they would reply to the digital navigator’s text message once they had completed the summary questionnaire.

The client asked for the digital navigator to send the reminder on a specific day and time during the week, as this was suited to their schedule. This also allowed the client to plan by setting aside time dedicated to completing the questionnaire. The client thanked the digital navigator, commenting that they had found the coaching session to be very helpful and that they felt more confident using the Innowell platform in the future.

**Summary of ongoing contact**

After the coaching session, the digital navigator let the psychologist know that the client had completed the full questionnaire and informed them of the accountability plan. The client and digital navigator also scheduled a second Zoom session, for a month later.

**Outcome**

This plan was successful for 4 weeks, in that the client remained engaged with Innowell and completed the summary questionnaire when reminded by the digital navigator. The client was also responsive to the digital navigator’s text messages and would reply as agreed.

However, the client disengaged with the digital navigator’s reminders and stopped using Innowell after this 4-week period. The digital navigator kept the clinician informed and let them know when the client had disengaged. The client missed the second Zoom with the digital navigator and informed the clinician of the missed appointment.

The client reengaged with Innowell 3 months later, for 2 entries, before disengaging again. The client did not reach out directly to the digital navigator during their reengagement.

Textbox 2. **Case study 2: Value of troubleshooting and improving comfortability.**

**Client details**

The client had been using Innowell on-and-off with their psychiatrist for approximately 2 years.

**Initial session with digital navigator**

The client said they had been trying to use the platform as best as they could, but they were uncertain if they were using it correctly and asked the digital navigator, “how do I re-do it? I don’t know what to do, so a lot of it [using Innowell] is through my own initiative.” They also wanted to provide feedback regarding the functionality of the platform.

Although their psychiatrist was encouraging and incorporated the technology during some sessions, very minimal instructions were provided to them at the start on how to use the technology, and the client noted that it was “not explained to me on how to use it.” The client had previously asked the administrative staff at the service for instructions but “they didn’t know either.”

The client had been completing the full questionnaire each time they engaged with the platform but said this was not always possible due to other commitments and they did not always have the time (as it can take 30 minutes to complete).

The digital navigator showed the client how to access the shorter summary questionnaire on the personalized home dashboard, which takes approximately 5 minutes to complete, and explained they could access more specific questionnaires by clicking on individual health cards on the dashboard.

The client provided feedback to the digital navigator on how they thought other people might be less likely to persevere and engage with DMHT, like Innowell, if it was implemented in a “wish-washy way by practices.”

The client reflected that, while not always discussed or reviewed at every session, their psychiatrist’s uptake of Innowell was an encouraging factor for their own use. The client also saw the value and benefits that routine outcome monitoring provided them with managing their own symptoms.

At the end of the coaching session, the client told the digital navigator, “It would have been really helpful to have you at the start to help set up the profile. There’s a lot of words to understand in the full questionnaire, how does someone understand who might be suffering severely? I think it would be good to have a mentor for this. Having someone sit with you in person or online would be amazing, it’s been really helpful.”

**Outcome**

This client continues to use Innowell every 1-2 weeks.

### Mutually Beneficial Relationships Between the Health Professionals, Digital Navigator, and Client

Similarly, the benefits of the DMHT for measurement-based and personalized care are dependent on the strength of collaboration between health professionals, digital navigators, and clients. When all 3 parties worked together to create shared goals for using the DMHT and to troubleshoot barriers, the perceived benefits of the platform were greater.

In one instance described in case study 3 (Textbox 3), both the health professional and the client reported that they had previously stopped discussing the platform during sessions because they believed that the other party was not interested in the data, without addressing why this was the case. They were not necessarily avoiding discussing the technology but did not see it as a priority during sessions when other problems needed to be discussed.

In this case, the digital navigator was able to empower the client to raise their concerns with their health professional and was able to provide simple solutions that helped to integrate the DMHT into care, ensuring that the client was using Innowell appropriately. It is important that health professionals and clients make shared decisions about how and why DMHTs are going to be used in care. Digital navigators can enhance this process by explaining the intended purpose, improving trust, and troubleshooting common barriers. This case study shows that the digital navigator can effectively empower clients to have specific discussions with health professionals about how they would like to use DMHTs.

Clients use Innowell for outcome measurement, yet this requires health professionals to review and respond to the data provided by clients. As shown in case study 4 (Textbox 4), some clients regularly completed platform data because they had been asked to by health professionals at the beginning of treatment, yet became frustrated when the data were not discussed during sessions. This suggests that health services should not simply introduce DMHT without providing adequate support to fully integrate them into care review processes. The time and effort for clients to engage with the digital navigator and platform may actually be unrealized if health professionals are not utilizing the information provided to guide personalized decision-making.

Taken together, our experiences suggest that the best way to implement a digital navigator is to make sure that they are seen as part of the care team and are helping to improve traditional mental health treatments alongside health professionals. Health professionals and clients will need assistance to build trust and comfortability with using new digital tools. Thus, the biggest improvements to mental health treatments are likely to be seen when health professionals, digital navigators, and clients are working together to integrate DMHT into care processes, and making joint decisions about the goals for using this technology and how barriers will be overcome.

Textbox 3. **Case study 3: The digital navigator can strengthen shared decision-making between health professionals and clients.**

**Initial session with the digital navigator**

The client met with the digital navigator in person. They were familiar with the purpose of Innowell (routine outcome monitoring and self-tracking) but found it difficult to stay motivated due to their clinician’s lack of engagement with reviewing Innowell, despite the clinician’s encouragement to use Innowell. The client also explained that they found it burdensome to complete the full questionnaire.

The client said they had not been shown how to use the platform in an ongoing capacity and explained that they had lost interest in dedicating the time to complete Innowell as their health professional was not reviewing or discussing it during their appointments, saying, “it was difficult to find half an hour to dedicate to answering Innowell and [health professional] weren’t looking at it anyway, it’s like what’s the point then?” The client explained they felt disheartened by this as they had been putting in effort to complete the full questionnaire prior to appointments, and they said they were frustrated that “[Clinician] asks me the same questions as Innowell does, if they looked at it beforehand, that would be better for me. It would be a better use of my appointments too, because [clinician] would already know how I am, or if they reviewed it with me at least.”

The digital navigator explained and showed the client that they can complete the shorter summary questionnaire rather than the full questionnaire each time.

**Outcome**

The client and digital navigator developed a plan for reminding the client to complete the summary questionnaire 2 days prior to their appointments and informing the clinician to review Innowell before and during this client’s appointments.

In the 8 months since this meeting, the client has maintained consistent use of the platform, with at least 16 entries.

Textbox 4. **Case study 4: Clients prefer the digital navigator to work in partnership with health professionals, not separately.**

**Client details**

Client had previously met with the digital navigator for a coaching and onboarding session. After some time had passed, the client asked the digital navigator for online resources, specifically to help with studying, reduce procrastination, and improve organizational skills and habits.

**Session with digital navigator**

The client and digital navigator met over Zoom for approximately 1 hour. The client was engaged with their Innowell and had completed the summary questionnaires regularly (fortnightly to monthly) to coincide with appointments.

However, the client explained that their clinician was not engaged with Innowell and it was not discussed during sessions.

After speaking with the digital navigator, the client commented that, while useful, Innowell requires support from both the clinician and a digital navigator to optimize the client’s care and use of the platform.

“Because I’m talking to you, it’s very helpful. But Innowell alone isn’t very helpful because it’s done by itself.”

The digital navigator discussed and suggested some digital and online resources with the client that could assist with their goals (as mentioned in the “Client details” section). At the conclusion of the Zoom call, the client said they found meeting with the digital navigator to discuss their Innowell and find other digital online resources to be very helpful, as was the digital navigator’s encouragement of their continued engagement with Innowell.

**Summary of ongoing contact**

The digital navigator provided feedback to the service about the client’s use of Innowell and their request for more support with studying and groups.

**Outcome**

The client continues to engage with Innowell, although it is unclear whether their clinician has begun incorporating Innowell into the client’s appointments. The client has completed the summary questionnaire 4 times over a 4-month period since the session with the digital navigator.

The client continues to seek additional support from the service for studying and groups. The client continues to use Innowell in the hope that their clinician will start to engage with the platform to provide the more personalized support they are seeking.

## Providing Virtual Support to Increase Feasibility and Accessibility of the Digital Navigator Role

Our experiences suggest that having a digital navigator “on-site” does not necessarily improve the value of this role, and that having a remote presence may help to improve both feasibility and acceptability of the role. Initially, the service requested that the digital navigator be available in person in a central area, such as the waiting room. This had a number of purposes including: (1) to prompt clients to complete questionnaires on the platform while waiting for their appointment to start; (2) to provide a visual reminder for health professionals and clients to use Innowell; and (3) to provide more opportunities for organic and ad hoc interactions between the digital navigator and health professionals (service staff reported that they did not have time for formal scheduled meetings with the digital navigator).

However, in practice, this did not produce the intended benefits. Health professionals were frequently off-site or unable to leave their consultation rooms, meaning they did not have time for ad hoc interactions with the digital navigator. In most cases, the health professional did not walk their client back out to reception, limiting opportunities for joint face-to-face interactions with the digital navigator. Moreover, some health professionals worked off-site through telehealth, or worked predominantly in an outreach setting. Similarly, in the wake of the COVID-19 pandemic, patients were more likely to attend sessions via telehealth (ie, phone or Zoom), meaning the benefits of face-to-face interactions could not be realized. Ultimately, we found that referrals were most likely to occur via health professionals providing the digital navigator with a list of names to follow-up with via phone or Zoom. This also allowed the digital navigator to contact clients at a time that was convenient to the client. Thus, given the busy nature of health clinics and the time commitment that clients are already making to attend appointments, there are clear advantages to providing remote digital navigation support.

## Discussion

Digital mental health technologies have significant potential to improve personalized and measurement-based care in mental health services, but they have struggled to realize this potential due to persistent challenges of implementing novel tools in real-world settings [6-8]. This paper explored the value of implementing a new role in services, a “digital navigator,” to improve the uptake and quality use of a novel multidisciplinary assessment platform, Innowell.

Digital mental health tools give clients a voice in their care. Digital Navigators ensure that voice is heard. However, a coordinated relationship between the health professional, client, and Digital Navigator has shown the clearest indicators of success in engagement and uptake of these tools.

[CG, digital navigator]

Overall, we found that the digital navigator was particularly useful when they were introduced to clients by health professionals as a member of the care team and assisted with troubleshooting problems and demonstrating the functionalities of the platform. By contrast, when health professionals had not embraced Innowell into their day-to-day practices, had limited interactions with the digital navigator, and did not regularly discuss Innowell data with clients during care, clients were unlikely to continue engaging with Innowell or the new support role. Therefore, digital navigators should not provide a stand-alone service to clients or health professionals but should instead work closely with both parties to enhance existing mental health treatments. Our observations suggest that, when this occurs, the quality of the care experience increases through greater transparency and shared decision-making.

This case study contributes to existing literature by providing practical recommendations from real-world experiences. There are limited guidelines or protocols that inform how digital navigator roles should be implemented in real-world settings [12,15,16,24]. Previous research has predominantly focused on using digital coaching to support self-guided online modules, and only 3 previous studies have explored the potential value of using peer workers to support digital transformation of services [15]. By contrast, we focused on implementing a measurement-based DMHT that is focused on tracking multidimensional client outcomes and assisting with more personalized and efficient treatment planning [26]. In addition, a peer worker added significant value to the role because they were seen as partners and advocates by clients. This enabled the digital navigator to identify how each client was most likely to benefit from the DMHT and to make shared decisions with them and their health professionals about how the DMHT could add value.

Our experiences also have some broader implications for the implementation of new DMHTs in mental health services. Successful implementation of DMHTs is reliant on health services creating optimal environments for digital transformation to occur. Noel and colleagues [14] theorized that digital navigators would help “to enhance the therapeutic bond between the client and the clinician” by working with both parties to identify recovery goals that will be the focus of monitoring, and by ensuring that all interactions are focused on these goals; this would ultimately improve clients’ “sense of control over their mental health.” Consistently, our experiences demonstrate that the relationship between the health professional, client, and digital navigator is mutually beneficial. When health professionals integrated Innowell into care and made shared decisions about the goals for using the platform with the client and the digital navigator, clients reported having more positive experiences and more frequent engagement with the platform. Thus, while these roles have predominantly been implemented to assist with recommending self-help apps used outside of therapy, health professionals and clients alike may receive more value from DMHT when they are used to enhance existing care.

Despite these contributions, our paper also had several limitations. Importantly, our findings were based on observational data recorded by the digital navigator themselves. As such, they are likely to be biased by the digital navigator’s own experiences, attitudes, and reflections. Thus, we have presented our experiences as an observational case study because of the need to improve transparency and guidelines about how digital navigator roles are being implemented. However, future research should focus on more detailed evaluations that will serve as a more robust exploration of the value and effectiveness of this role [20]. Relatedly, the digital navigator only worked with a small number of health professionals and clients at the health service. Future work should focus on quantifying in more detail the time investment and costs associated with this role to better understand the utility and scalability in health services. There is also scope for future research into how the role could additionally be used to facilitate and support fidelity to a range of intervention approaches (eg, cognitive behavioral therapy). Communication with the digital navigator was voluntary; thus, those who did interact with them were most likely to view this role favourably. As such, longitudinal large cohort research in real-world settings is needed to fully understand the benefits of this role.

Taken together, our experiences suggest that when a digital navigator works closely alongside health professionals and clients to create goals for new DMHTs, provide encouragement to engage with the tool, build on users’ skills to increase accessibility and enhance confidence, and provide technical troubleshooting, clients better understand the purpose of the technology, are more likely to complete ongoing assessments, and are more likely to see value from using the DMHT in their care. Thus, digital navigators have an important place in the digital transformation of health services but should not be seen as a stand-alone role.

## Abbreviations

DMHT: digital mental health tools
EMPOWERED: Educate, Measurement-based, Personalised, Openness, Work collaboratively, Engage, Recovery, Enhanced Digitally
